# Advanced Diffuse Systemic Sclerosis With Progressive Esophageal Dysfunction: Clinical Challenges in a Public Hospital

**DOI:** 10.7759/cureus.97255

**Published:** 2025-11-19

**Authors:** Carlos Solórzano Flores, Abner Baquedano, Allan Daniel Maradiaga Mejía, Juan José Flores

**Affiliations:** 1 Faculty of Medical Sciences, Universidad Nacional Autónoma de Honduras, Tegucigalpa, HND; 2 Faculty of Medicine and Surgery, Universidad Católica de Honduras, Tegucigalpa, HND; 3 Department of Internal Medicine, Hospital San Felipe, Tegucigalpa, HND

**Keywords:** anti-scl-70 antibodies, diffuse systemic sclerosis, gastrointestinal complication, micro-vasculopathy, superinfection

## Abstract

Diffuse systemic sclerosis (SS) is a rare autoimmune connective tissue disorder characterized by widespread fibrosis, vasculopathy, and autoantibody production. Gastrointestinal involvement, particularly esophageal dysfunction, is among the most frequent and morbid manifestations. Diagnosing and managing these complications can be especially challenging in advanced disease stages and in resource-limited settings, where specialized investigations and treatments may be inaccessible. We report a 34-year-old woman with diffuse SS who developed rapidly progressive oral intolerance, severe malnutrition, and superinfected cutaneous ulcerations. Barium esophagography demonstrated markedly delayed esophageal clearance, consistent with advanced esophageal dysmotility. Esophageal manometry and endoscopy could not be performed due to patient intolerance and restricted oral opening. Despite aggressive management including cyclophosphamide, targeted antibiotic therapy, and supportive care, the patient’s condition deteriorated, culminating in multi-organ failure secondary to septic shock. This case illustrates the aggressive and life-threatening nature of gastrointestinal involvement in diffuse SS. It underscores the diagnostic and therapeutic challenges faced in resource-constrained environments and highlights the critical importance of early recognition, vigilant supportive management, and adaptation of evidence-based recommendations to local healthcare realities.

## Introduction

Systemic sclerosis (SS), or scleroderma, is a chronic autoimmune disease characterized by fibrosis of the skin and internal organs, small-vessel vasculopathy, and autoantibody presence [1-5]. Based on cutaneous involvement, SS is classified as diffuse, limited (CREST syndrome: calcinosis, Raynaud phenomenon, esophageal dysmotility, sclerodactyly, and telangiectasia), or sine scleroderma, the latter lacking cutaneous manifestations [6-8]. Clinical features include progressive skin thickening, variable facial involvement, telangiectasias, hyperpigmentation, and calcifications. Raynaud’s phenomenon, often accompanied by pain and digital ulcers, is a hallmark, while arthralgias and internal organ involvement are frequent.

The global incidence of SS is estimated at 4-12 cases per million per year, with a prevalence ranging from 50-300 per million, predominantly affecting women between 30 and 50 years of age [5,7,8]. Gastrointestinal (GI) involvement is highly prevalent, affecting up to 90% of patients, and is a major cause of morbidity [9-11]. Esophageal dysfunction arises from smooth muscle atrophy and replacement by fibrotic tissue, leading to lower esophageal sphincter hypotonia, loss of peristalsis, and chronic reflux. These changes predispose to dysphagia, aspiration, and malnutrition [11]. The pathophysiological link between vascular and fibrotic changes, mediated by endothelial injury and fibroblast activation, explains the progressive nature of dysphagia and the co-occurrence of vasculopathy and gastrointestinal symptoms [12].

Despite its clinical significance, management options are often limited, especially in low-resource settings, and remain largely supportive. We report a case of advanced diffuse SS with rapidly progressive GI involvement, highlighting diagnostic challenges, severe esophageal dysfunction, and a fatal outcome.

This case exposes the severe, rapidly progressive gastrointestinal decline in diffuse systemic sclerosis, emphasizing the fatal consequences of delayed diagnosis and the need for adaptable care strategies in challenging clinical contexts.

## Case presentation

A 34-year-old female from eastern Honduras, with no significant past medical history, presented with an eight-month history of progressive joint pain involving the shoulders, elbows, and knees, accompanied by generalized weakness and restricted range of motion.

Physical examination revealed facial tightening, microstomia, xerostomia, Raynaud’s phenomenon, sclerodactyly, calcinosis of the upper extremities, and a modified Rodnan skin score of 3/3 in the arms, forearms, abdomen, thighs, and legs, indicating diffuse cutaneous involvement.

The initial laboratory evaluation (Table 1) showed mild anemia, elevated inflammatory markers, with positive anti-Scl-70 and negative anticentromere and anti-DNA antibodies.

**Table 1:** Initial laboratory evaluation ESR: Erythrocyte sedimentation rate

Parameter	Result	Reference Range
Leukocytes	9,880/µL	4,000–11,000/µL
Hemoglobin	11.2 g/dL	12–16 g/dL
Creatinine	0.3 mg/dL	0.6–1.1 mg/dL
Blood urea nitrogen	9.67 mg/dL	7–20 mg/dL
Glucose	88 mg/dL	70–99 mg/dL
Aspartate aminotransferase	38 U/L	<40 U/L
Alanine aminotransferase	43 U/L	<40 U/L
C-reactive protein	24 mg/L	<3 mg/L
ESR	53 mm/h	<20 mm/h
Rheumatoid factor	<14 IU/mL	<14 IU/mL
Anti-Scl-70	Positive	Positive or Negative
Anticentromere	Negative	Positive or Negative
Anti-DNA	Negative	<30 IU/mL

Nailfold capillaroscopy demonstrated a late sclerodermiform pattern. Based on these findings, a diagnosis of diffuse systemic sclerosis was established. Initial treatment included mycophenolate mofetil 2 g/day, methotrexate 17.5 mg weekly, prednisone 5 mg/day, nifedipine 10 mg/day, and folic acid 5 mg/day.

**Figure 1:**
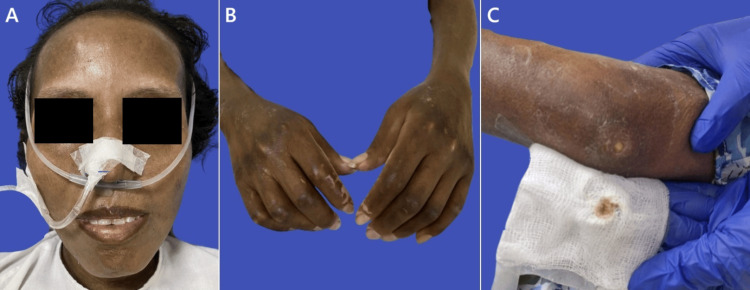
Physical examination findings in diffuse systemic sclerosis. (A) Facial involvement showing absence of wrinkles, reduced facial expression, and microstomia. (B) Sclerodactyly affecting the hands. (C) Superinfected ulceration on the left elbow.

After seven months, the patient developed progressive dysphagia, initially to solids and later to liquids, accompanied by severe asthenia, adynamia, abdominal pain, and impaired ambulation. Examination revealed marked malnutrition and superinfected ulcerations on both elbows (Figure 1).

On admission (day 0), height was 1.68 m, weight 43.03 kg, BMI 15.24 kg/m², and laboratory tests indicated worsening anemia and elevated inflammatory markers (Table 2).

**Table 2:** Admission laboratory findings ESR: Erythrocyte sedimentation rate

Parameter	Result	Reference Range
Leukocytes	8,650/µL	4,000–11,000/µL
Hemoglobin	9.7 g/dL	12–16 g/dL
Creatinine	0.3 mg/dL	0.6–1.1 mg/dL
Blood urea nitrogen	9.7 mg/dL	7–20 mg/dL
Glucose	123 mg/dL	70–99 mg/dL
Aspartate aminotransferase	41 U/L	<40 U/L
Alanine aminotransferase	41 U/L	<40 U/L
C-reactive protein	192 mg/dL	<3 mg/L
ESR	85 mm/h	<20 mm/h

Ulcer cultures grew Enterococcus group D (sensitive to vancomycin and ciprofloxacin) in the left arm and Acinetobacter baumannii (sensitive to imipenem and ceftazidime) in the right arm.

In line with standard management of severe diffuse disease and due to worsening skin and gastrointestinal involvement despite prior dual immunosuppression, treatment with intravenous cyclophosphamide (1 g monthly) was initiated, with a planned duration of three months, combined with oral prednisone (10 mg every 8 hours for 15 days), gradually tapered to 5 mg/day. Mycophenolate and methotrexate were discontinued to minimize toxicity.

Nutritional support was initiated via nasogastric tube to maintain caloric intake, as parenteral nutrition was unavailable due to financial and resource limitations. Proton pump inhibitors and prokinetic agents were administered to reduce reflux and improve gastric emptying.

**Figure 2:**
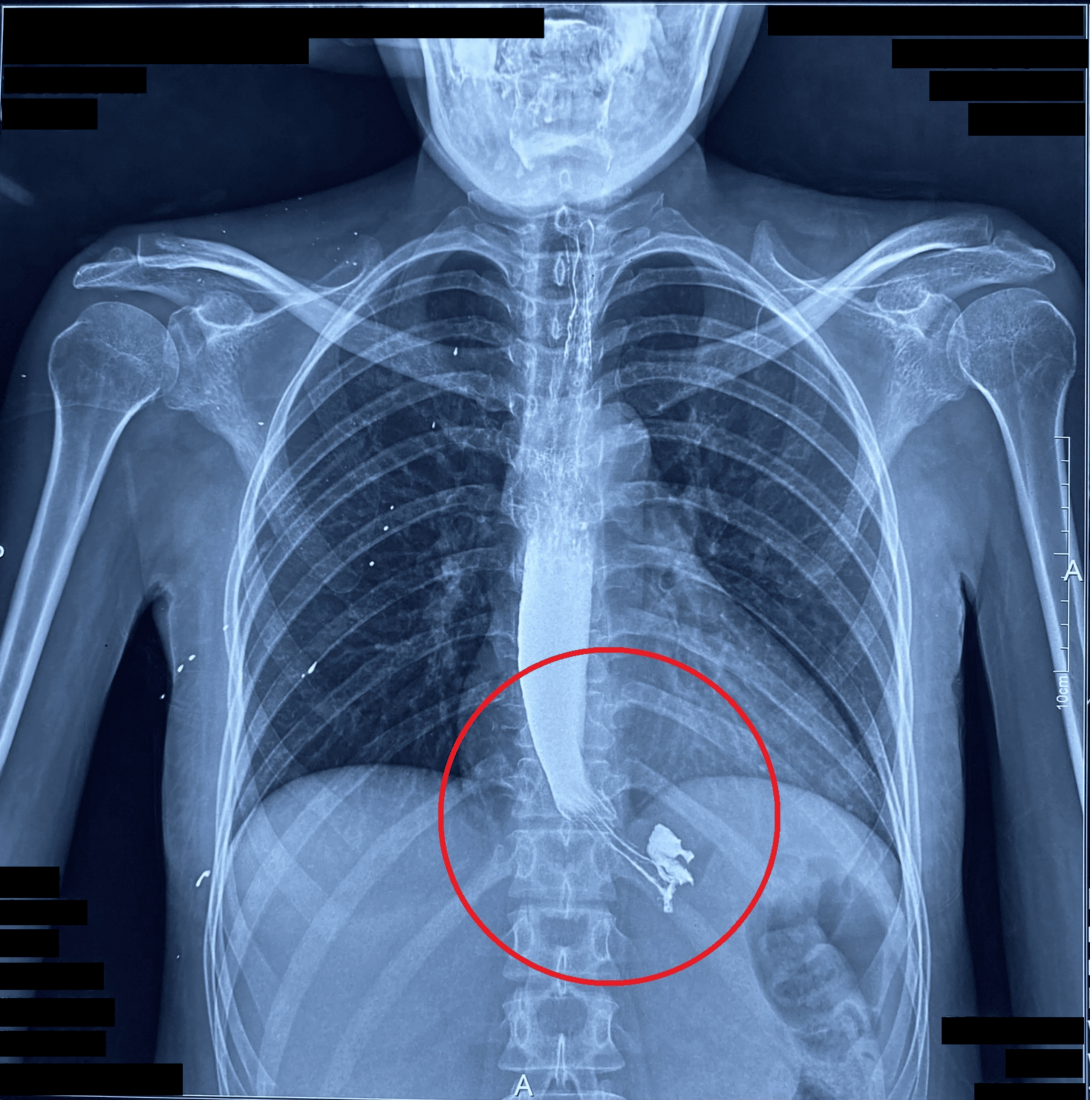
Barium esophagogram performed 10 minutes after contrast administration showing persistent contrast in the distal esophagus (red circle), consistent with severe esophageal dysmotility

A barium esophagogram performed 10 minutes after contrast ingestion demonstrated markedly delayed esophageal clearance with retained contrast in the distal third, consistent with advanced esophageal dysmotility (Figure 2). This finding reflects advanced smooth muscle fibrosis and functional obstruction typical of diffuse systemic sclerosis.

Esophageal manometry and endoscopy were not performed due to patient intolerance and limited oral opening, respectively. Gastrostomy was considered, but the patient’s critical condition rendered the procedure unsafe.

Despite targeted antimicrobial therapy and supportive measures, she developed septic shock with renal and respiratory failure, culminating in multi-organ failure by hospital day 21.

## Discussion

SS is a rare autoimmune disease, with an incidence of 4-12 cases per million per year, predominantly affecting women in the fourth and fifth decades [7,8,13]. This patient’s age and sex align with epidemiological data. Pathogenesis involves interactions between genetic susceptibility and environmental triggers, leading to immune dysregulation, endothelial injury, and fibroblast-mediated fibrosis [14]. No triggers were identified in this patient, underscoring the heterogeneous and sometimes aggressive nature of diffuse SS. Disease severity is commonly assessed with the modified Rodnan skin score and nailfold capillaroscopy [10]. Our patient exhibited severe cutaneous involvement and a late sclerodermiform pattern, consistent with prior reports linking these findings to a higher risk of organ involvement [15,16].

GI involvement is among the most frequent internal complications, affecting up to 90% of patients [9,15]. Esophageal involvement occurs in 75-90% of cases, due to smooth muscle atrophy and fibrosis, resulting in a lack of peristalsis and sphincter incompetence. Clinically, this manifests as progressive dysphagia, reflux, and eventual nutritional failure [7]. In this case, the patient’s BMI of 15.24 kg/m² indicated severe malnutrition. Although serum albumin could not be measured, weight loss exceeded 20% over six months, reflecting profound catabolism. Malnutrition further compromised immune defense, predisposing to the superinfection of cutaneous ulcers and subsequent septic shock.

Barium studies suggested delayed clearance; however, manometry could not be performed, highlighting a limitation in advanced cases [16]. The clinical course reflects severe morbidity associated with GI involvement, comparable to prior reports showing malnutrition and aspiration risks in symptomatic patients [15,16].

Autoantibody profiling remains essential for diagnosis and prognosis. Anti-topoisomerase I (Scl-70) antibodies, present in this patient, are associated with diffuse SS and severe organ involvement [7]. Serial laboratory data demonstrated a CRP increase (24 to 192 mg/L) and ESR rise (53 to 85 mm/h), consistent with severe infection [7,10]. Antibiotic therapy was guided by sensitivity results, but the lack of intensive care facilities and parenteral nutrition limited further interventions. The absence of blood cultures, vasopressor support, and invasive monitoring reflects real-world constraints in public hospitals.

Optimal management includes immunosuppression, acid suppression, prokinetic therapy, and nutritional optimization [15,16]. Cyclophosphamide was selected as a second-line agent given the aggressive progression. However, real-world constraints, such as the absence of parenteral nutrition, endoscopy, or intensive monitoring, restricted comprehensive care. In similar settings, adjunctive measures such as metoclopramide or domperidone for motility, nutritional supplementation, and early multidisciplinary collaboration (rheumatology, gastroenterology, nutrition, infectious disease) may improve outcomes even when advanced tools are unavailable.

Previous reports describe similar cases of diffuse SS with severe GI involvement and malnutrition leading to fatal complications [9,15,16]. However, few have detailed the compounded effect of limited diagnostic and supportive resources, as demonstrated here. This underscores the urgent need for context-adapted protocols emphasizing early detection, prophylactic antibiotics for ulcer management, and nutritional support within existing public-health frameworks.

## Conclusions

Diffuse systemic sclerosis is a heterogeneous autoimmune disease in which gastrointestinal involvement is a major contributor to morbidity and, indirectly, mortality. In this case, esophageal dysfunction progressed rapidly within months of disease onset, leading to severe malnutrition that predisposed the patient to superinfection and septic shock. Although death resulted primarily from sepsis secondary to infected ulcers, the underlying nutritional failure and systemic immunosuppression created the conditions for this fatal outcome. Diagnostic and therapeutic limitations, particularly the unavailability of manometry, endoscopy, parenteral nutrition, and intensive care, illustrate the challenges of managing advanced systemic sclerosis in resource-poor settings. Earlier identification of dysphagia, timely initiation of nutritional support, and prevention of ulcer superinfection might have mitigated disease progression. Nasogastric feeding, though necessary, proved suboptimal in severe esophageal dysmotility, and earlier consideration of gastrostomy might have improved tolerance and caloric intake if the patient’s condition had allowed it. The emergence of multidrug-resistant organisms such as Acinetobacter baumannii underscores the importance of infection control and antibiotic stewardship in immunosuppressed patients. Ultimately, this case highlights the need for vigilant multidisciplinary management, context-adapted care strategies, and proactive prevention of nutritional and infectious complications to improve survival in diffuse systemic sclerosis within resource-limited healthcare systems.
